# Supplementary material for: The influence of spouses and their driving roles in self-regulation: A qualitative exploration of driving reduction and cessation practices amongst married older adults
Source: PLoS One. 2020 May 15;15(5):e0232795. doi: 10.1371/journal.pone.0232795 (PMC7228106; doi:10.1371/journal.pone.0232795)
Supplement: S1 File — (DOCX) [file pone.0232795.s002.docx]

**Supporting information**

**S1 File. Guided questions.**

**Guided questions for current car drivers**

1. Could you please share your feelings and experiences as a car driver?

- How old were you when you first driving?
- What does driving means to you?
- How important is being able to drive to you?

1. Has your driving changed over the past 5 years? How often do you drive now as compared to 5 years ago?

- Do you drive more/less/about the same?
- Do you make fewer trips?
- Do you make shorter/longer trips?
- Do you drive at a lower speed?
- Do you avoid driving when is not necessary? If yes, when would that be and why do you avoid?

1. Are there any problems that may influence your driving ability? Kindly elaborate further on these aspects.

- Are you facing any difficulties (medical conditions, physical changes, traffic, confidence, etc.) when you are driving?
- Do you have any concern about your driving?
- Have you ever concerned that your driving might be a risk to others?

1. Have any of your family members, friends or physicians expressed any concerns about your ability to drive safely?

- Elaborate on the concerns if there are any.
- Do their worries concern you?
- How do their concerns affect you?

1. Have you thought of stop driving someday? Do you think you will ever stop driving?

- In your opinion, what would be the events that would prompt you to consider stop driving?
- What would be the most difficult adjustments?
- What alternative options for transport did you think about?

1. Does your spouse drive too?

- How do you share driving?
- Is there anyone else in the household who can give you a ride?
- Who is the principal driver in the household?
- Does the presence of other drivers in the household increase the likelihood of reduce/stop driving?

1. What do you think about co-piloting/navigation?

- Would you prefer to have someone assisting you as a co-pilot/navigator whilst you drive?
- Have you ever experienced being one before?

1. If you have sufficient access to public transportations, would you utilise them to sustain your mobility needs when you quit driving?

- Can you think of any help/support you can benefit from during pre- or post-cessation?

1. What would be your advice to those who have difficulty driving and who are in the process of making the decision to reduce or stop driving?
   - Is there any advice that you would like to share?
2. Do you have any comments or suggestions that you would like to share?

**Guided questions for former car drivers**

1. Could you please share your feelings and experiences before and after you quit driving?

- How old were you when you first driving?
- How old were you when you stop driving?
- What did driving mean to you?
- How important was it to be able to drive?
- How did you feel about stopping driving at that time?
- How do you feel about the decision now?

1. Why did you stop driving?

- Were there any problems limiting your driving ability? Or any events leading up to the decision to stop driving?
- Have you ever concerned that your driving might be a risk to others?
- Did you plan ahead and gradually stop from driving?
- Was there a specific event that made you think about stopping driving (e.g., crash, near miss, medical condition, etc.)?
- Did your driving pattern change before making the decision (frequency of trips, distance travelled, speed and tendency to avoid)?
- Who did you talk to about the decision? Did you seek any professional advice?

1. Did your family or your doctor have any concerns about your ability to drive safely? What were the concerns of family members, friends or physician for you to drive? What did they say?
   - Elaborate on the concerns if there are any.

- Did their worries concern you? How did their concerns affect you?
- Did anyone influence your decision to stop driving or was it a personal decision?
- How did your family or your doctor raise any concerns with you?

1. What are the most important adjustments after you have decided to stop driving? Please elaborate.

- How did you cope with the transition? Are you coping well?
- Have there been changes to your daily routine?
- Do you think your ability to get to places has changed since giving up driving?

1. Does your spouse drive? Do you have anyone in the household who can drive?

- Who is the principal driver in the household?
  - Did the presence of other drivers in the household influence your decision to stop driving?

1. If you want to go somewhere, what would you do? What mode of transportation do you use now?

- Do you often rely on your spouse/others for transportation support?
- Have you ever felt reluctant to ask for transportation service from them?
- If you had a choice, what would be your preferred alternative options for transport?

1. What do you think about the role as a co-pilot/navigator? Have you ever experienced being one before?

- If you are still driving, would you prefer to have someone assisting you as a co-pilot/navigator whilst you drive?

1. If you have adequate access to public transportations, would you utilise them to sustain your mobility needs?

- Can you think of any help/support you would like to have during pre- and post-cessation?

1. What would be your advice to those who have difficulty driving and who are in the process of making the decision to reduce or stop driving?

- Is there any advice you would like to share?

1. Do you have any comments or suggestions that you would like to share?
