# Supplementary material for: The influence of spouses and their driving roles in self-regulation: A qualitative exploration of driving reduction and cessation practices amongst married older adults
Source: PLoS One. 2020 May 15;15(5):e0232795. doi: 10.1371/journal.pone.0232795 (PMC7228106; doi:10.1371/journal.pone.0232795)
Supplement: S1 Table — (DOCX) [file pone.0232795.s003.docx]

**Supporting information**

**S1 Table. Demographic characteristics and driving history of participants who had participated in this study.**

| Variables | All participants | Male partners *(Husband)* | Female partners *(Wife)* |
| --- | --- | --- | --- |
| Age, *Median (range)* | 68 (60-79) | 69 (60-79) | 64 (60-78) |
| 60-74 years | 17 (77.3) | 8 (72.7) | 9 (81.8) |
| >74 years | 5 (22.7) | 3 (27.3) | 2 (18.2) |
| Ethnicity |  |  |  |
| Chinese | 13 (59.1) | 6 (54.5) | 7 (63.6) |
| Indian | 6 (27.3) | 3 (27.3) | 3 (27.3) |
| Malay | 2 (9.1) | 1 (9.1) | 1 (9.1) |
| Others | 1 (4.5) | 1 (9.1) | 0 (0) |
| State |  |  |  |
| Selangor | 16 (72.7) | 8 (72.7) | 8 (72.7) |
| Kedah | 6 (27.3) | 3 (27.3) | 3 (27.3) |
| Level of education |  |  |  |
| Secondary | 14 (63.6) | 7 (63.6) | 7 (63.6) |
| Tertiary | 8 (36.4) | 4 (36.4) | 4 (36.4) |
| Work status |  |  |  |
| Full-time | 3 (13.6) | 0 (0) | 3 (27.3) |
| Part-time | 2 (9.1) | 1 (9.1) | 1 (9.1) |
| Retired | 16 (72.7) | 10 (90.9) | 6 (54.5) |
| Nor working | 1 (4.6) | 0 (0) | 1 (9.1) |
| Driving status |  |  |  |
| Continued driving | 20 (90.9) | 11 (100) | 9 (81.8) |
| Ceased driving | 2 (9.1) | 0 (0) | 2 (18.2) |
| Driving frequency ^a^ |  |  |  |
| Occasionally | 5 (25) | 1 (9.1) | 4 (44.5) |
| Almost every day | 9 (45) | 6 (54.5) | 3 (33.3) |
| Every day | 6 (30) | 4 (36.4) | 2 (22.2) |
| Driving distance ^a^ |  |  |  |
| Short distance | 19 (95) | 10 (90.9) | 9 (100) |
| Long distance | 1 (5) | 1 (9.1) | 0 (0) |
| Crash history within the past 5 years | | | |
| Yes | 9 (40.9) | 8 (72.7) | 1 (9.1) |
| No | 13 (59.1) | 3 (27.3) | 10 (90.9) |
| Self-reported medical history | | | |
| Hypertension | 5 (22.7) | 1 (9.1) | 4 (36.4) |
| Diabetes mellitus | 1 (4.5) | 1 (9.1) | 0 (0) |
| Heart problems | 1 (4.5) | 1 (9.1) | 0 (0) |
| Cataract or glaucoma | 4 (18.2) | 1 (9.1) | 3 (27.3) |
| Hearing problems | 2 (9.1) | 1 (9.1) | 1 (9.1) |
| Arthritis | 7 (31.8) | 3 (27.3) | 4 (36.4) |

^a^ *Current drivers only.*

*Note. All data are presented as frequency and percentage unless otherwise specified.*
